# Recent progress and applications of single-cell sequencing technology in breast cancer

**DOI:** 10.3389/fgene.2024.1417415

**Published:** 2024-09-18

**Authors:** Yousef M. Hawsawi, Basmah Khoja, Abdullah Omar Aljaylani, Raniah Jaha, Rasha Mohammed AlDerbi, Huda Alnuman, Mohammed I. Khan

**Affiliations:** ^1^ Research Center, King Faisal Specialist Hospital and Research Center, Jeddah, Saudi Arabia; ^2^ Department of Biochemistry and Molecular Medicine, College of Medicine, Al-Faisal University, Riyadh, Saudi Arabia

**Keywords:** single-cell sequencing, breast cancer, tumor heterogeneity, microenvironment, personalized therapy

## Abstract

Single-cell RNA sequencing (scRNA-seq) technology enables the precise analysis of individual cell transcripts with high sensitivity and throughput. When integrated with multiomics technologies, scRNA-seq significantly enhances the understanding of cellular diversity, particularly within the tumor microenvironment. Similarly, single-cell DNA sequencing has emerged as a powerful tool in cancer research, offering unparalleled insights into the genetic heterogeneity and evolution of tumors. In the context of breast cancer, this technology holds substantial promise for decoding the intricate genomic landscape that drives disease progression, treatment resistance, and metastasis. By unraveling the complexities of tumor biology at a granular level, single-cell DNA sequencing provides a pathway to advancing our comprehension of breast cancer and improving patient outcomes through personalized therapeutic interventions. As single-cell sequencing technology continues to evolve and integrate into clinical practice, its application is poised to revolutionize the diagnosis, prognosis, and treatment strategies for breast cancer. This review explores the potential of single-cell sequencing technology to deepen our understanding of breast cancer, highlighting key approaches, recent advancements, and the role of the tumor microenvironment in disease plasticity. Additionally, the review discusses the impact of single-cell sequencing in paving the way for the development of personalized therapies.

## Graphical Abstract

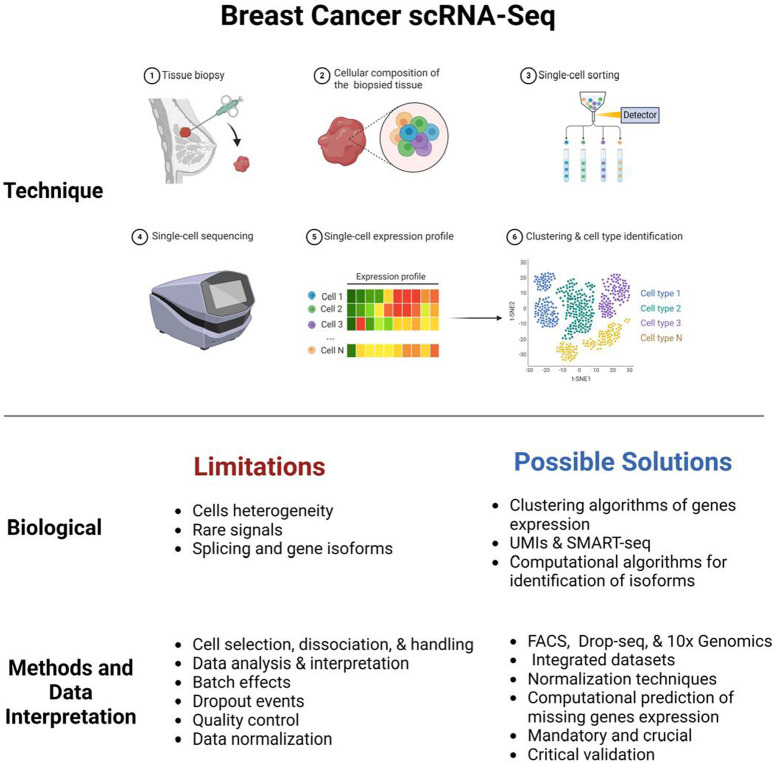

## 1 Overview of breast cancer

Breast cancer (BC) is the most prevalent malignancy worldwide and the leading cause of cancer-related deaths (Katsura et al., 2022). It is a complex disease characterized by cellular heterogeneity across genomic, transcriptomic, and proteomic levels, with a diverse range of subtypes and molecular profiles that pose significant challenges for diagnosis and treatment. The heterogeneity within solid tumors, which consist of various cell types and clonal subpopulations, complicates molecular classification and hinders the accurate assessment of clinical samples. This complexity is further aggravated by genetic variations, such as ERBB2 gene amplification and aneuploidy, which significantly influence therapeutic decision-making and patient outcomes (Barzaman et al., 2020). The intricate nature of breast cancer also impacts detection, progression, and predication of therapeutic efficacy. The two primary types of BC are ductal carcinoma *in situ* and invasive carcinoma, with the latter further classified into subtypes such as luminal A, luminal B, HER2-enriched, and triple-negative BC (TNBC). TNBC, which accounted for 15% of all BCs as of 2018, is the most aggressive subtype, associated with poorer long-term outcomes compared to other types. Single-cell genomic methods have emerged as transformative tools to address these challenges by enabling the profiling of scarce cancer cells, monitoring of circulating tumor cells (CTCs), and detecting rare resistant clones in clinical samples. These applications are expected to advance the understanding of BC at the single-cell level and improve its clinical management (Navin and Hicks, 2011).

### 1.1 Current challenges in BC research

BC research faces several significant challenges, including treatment resistance, disease recurrence (Hawsawi et al., 2016), and limited understanding of tumor heterogeneity. Traditional bulk tissue genomic analysis lacks the resolution necessary to fully grasp the intricate interaction between different genetic events within the cellular hierarchy of tumors (Semlali et al., 2018). This limitation hampers a comprehensive understanding of disease initiation, evolution, relapse, and metastasis. Moreover, cellular heterogeneity–driven by aberrant stem cell proliferation and dynamic genomic profiles–presents a substantial obstacle to the development of effective targeted therapies (Hawsawi et al., 2018), as highlighted by Ye et al. (2016). The dynamic genomic landscape of BC contributes to immune evasion and resistance to chemotherapy (Barnawi et al., 2022). Another critical challenge in breast cancer research is the scarcity of reliable biomarkers for early detection and treatment monitoring. Despite the identification of several potential biomarkers, their clinical application is often constrained by issues of sensitivity and specificity. This variability impedes accurate predictions of disease progression and therapeutic outcomes, complicating the development of personalized treatment plans. Addressing this challenge underscores the need for advanced technologies, such as single-cell sequencing, which can provide detailed insights into clonal structures, capture spatiotemporal cell interactions, and identify rare cellular populations essential for understanding metastatic disease, drug resistance, and overall disease progression (Wills and Mead, 2015).

### 1.2 Single-cell sequencing technology: an introduction

Single-cell sequencing (SCS) technology has revolutionized the study of cellular heterogeneity by providing insights into the transcriptomic, genomic, epigenomic, and proteomic profiles of individual cells. SCS enables the evaluation of disease-state changes and offers a deeper understanding of tumors with diverse morphological and phenotypic characteristics (Mustachio and Roszik, 2022). Therefore, it is important to highlight recent advancements in molecular genetics.

Conventional next-generation sequencing, often referred to as bulk sequencing, has advanced considerably in recent years. The term “bulk sequencing” distinguishes it from single-cell techniques, which differ from standard DNA sequencing methods where DNA content of thousands to millions of cells is homogenized. In contrast, single-cell sequencing technologies allow for the examination of individual cells, enabling a more detailed and comprehensive analysis of genetic, epigenetic, and transcriptomic variations within a heterogeneous cell population. This shift from bulk to single-cell approaches has significantly enhanced our ability to uncover and understand the intricacies of cellular diversity and molecular processes at the individual cell level (Evrony et al., 2021).

**FIGURE 1:**
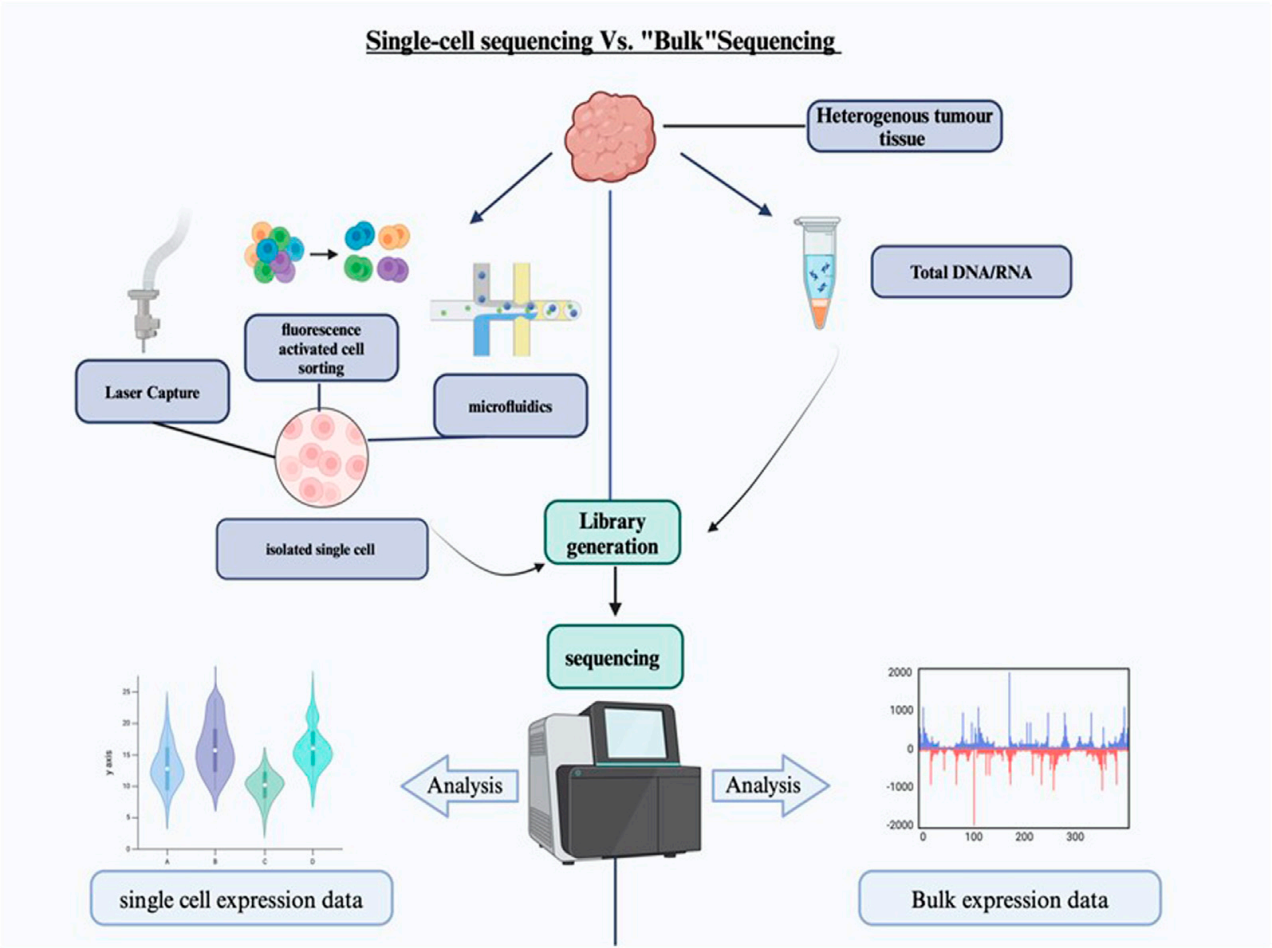
Single-cell sequencing vs. Bulk sequencing. The diagram shows the comparison between the single cell sequencing and the bulk sequencing. The heterogeneous tumor tissue is separated and sorted into single cells before the library generation. The data expression is more specific in single-cell sequencing.

A key advantage of SCS over bulk sequencing is its ability to detect rare cellular subpopulations that might otherwise be missed. These rare cells can play crucial roles in disease progression, drug resistance, and other critical biological processes. Figure 1 illustrates the differences between SCS and bulk sequencing. Single-cell technologies are primarily employed to measure the genome (scDNA-seq), DNA methylome, or transcriptome (scRNA-seq) of each cell within a population. These advanced techniques have proven invaluable in identifying novel mutations in malignant cells, evaluating tumor heterogeneity at the single-level, and investigating the dynamic variations in the epigenome during embryonic development (Evrony et al., 2021; Hu et al., 2023).

### 1.3 Methodologies for SCS

The pioneering experiment using single-cell mRNA sequencing took place in 2009, followed by the first single-cell DNA sequencing in human cancer cells in 2011, and the first single-cell exome sequencing in 2012 (Tang et al., 2009). Recent advancements in genetic sequencing technologies have further refined SCS, enabling scientists to map a cell’s genome, transcriptome, and other multiomics. These technologies not only reveal variations between individual cells but also uncover evolutionary connections among them. The term “SCS technologies” refers to the process of sequencing a single cell’s genome or transcriptome to obtain genomic, transcriptomic, or other multiomics data. This data is invaluable for identifying changes between different cell populations and their interactions over time. Compared to traditional sequencing technologies, single-cell technologies offer significant advantages, such as identifying small numbers of cells, uncovering heterogeneity among individual cells, and constructing detailed cell maps (Wen and Tang, 2018).

**FIGURE 2:**
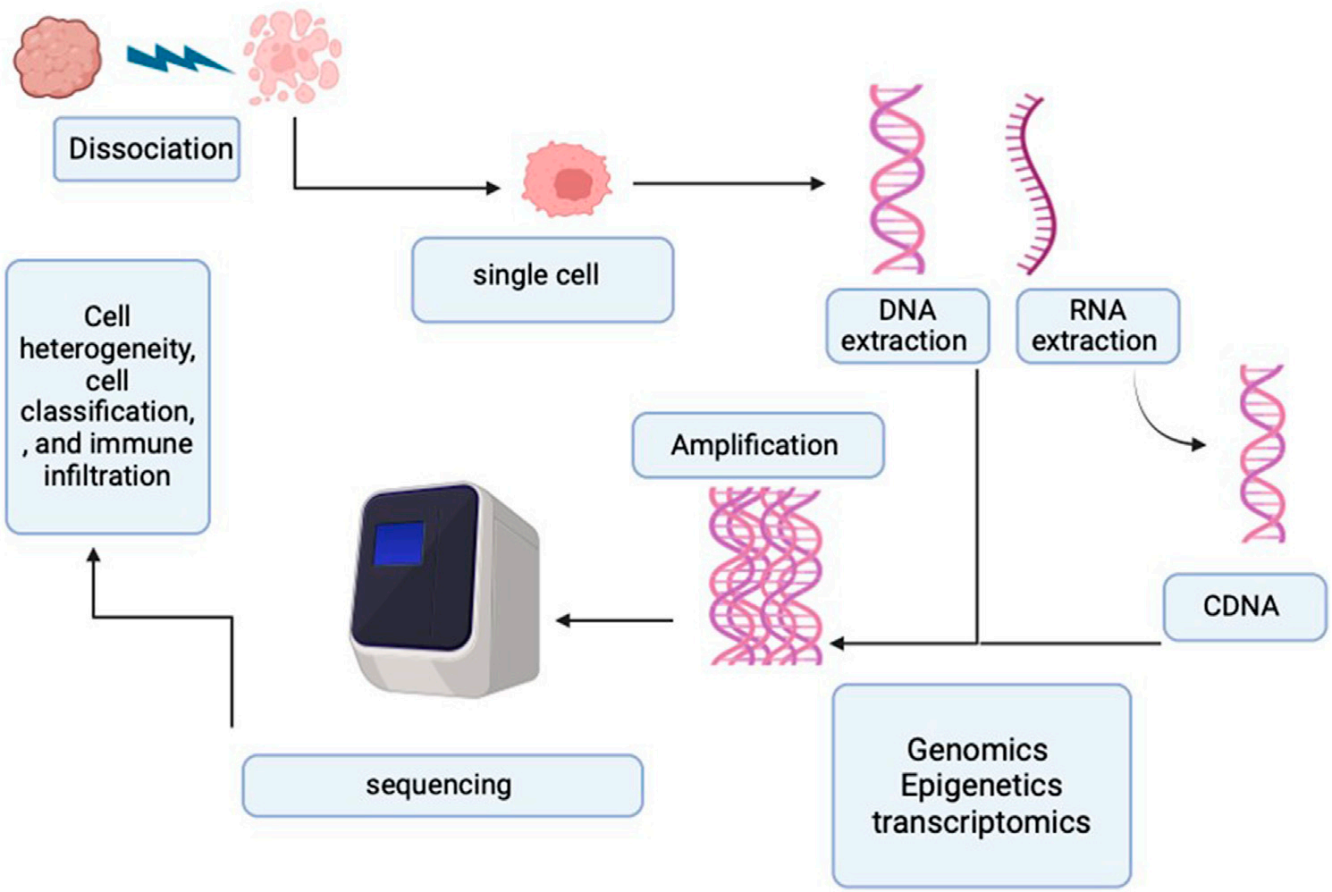
Process of single-cell sequencing. The diagram represents the single-cell process starting from the single-cell sorting and then the DNA/RNA extraction. Followed by the amplification and sequencing.

Initially, the adoption of SCS was limited due to its high cost and technical challenges. However, as technology advanced, numerous novel SCS techniques were developed, lowering the cost barrier and expanding its accessibility. Figure 2 illustrates the SCS process.

SCS technology employs various methodologies to evaluate heterogeneity in cellular populations at different molecular levels. For transcriptomic analysis, single-cell RNA sequencing (scRNA-seq) has become a crucial method for assessing the transcriptomic status of specific single-cell populations. Technologies such as Smart-seq, Smart-seq2, Quartz-Seq, and CEL-seq enable the measurement of mRNAs isolated from individual cells, allowing for the simultaneous barcoding and processing of thousands of single cells. This approach has been instrumental in uncovering cellular states, gene networks, and tumor transformations across a large number of individuals with colorectal cancer, demonstrating its potential for analyzing interacting cellular programs (Mustachio and Roszik, 2022).

Beyond transcriptomic analysis, single-cell DNA sequencing (scDNA-seq) techniques, including BGI, nuc-seq, SNES, and multiple annealing and loop-based amplification cycles (MALBAC), provide comprehensive genome-wide copy number profiles of individual cells, facilitating the detection of base-level mutations. Recent transposition-based methodologies, such as DLP and DLP+, address coverage and polymerase bias limitations, contributing to a better understanding of tumor cell copy number mutations and the development, progression, and evolution of BC (Twigger and Khaled, 2021; Alanazi et al., 2021).

Various techniques such as SCRB-seq, CEL-seq2, MARS-seq, Drop-seq, Smart-seq1, Smart-seq2, and 10 × Genomics are commonly used for scRNA-seq, each offering unique advantages depending on the number of cells analyzed and the level of noise in mRNA quantification. These advancements in SCS techniques have significantly expanded our understanding of cellular dynamics in BC (Hawsawi et al., 2022a), offering substantial promise for precision oncology and therapeutic development. Combined with other single-cell methodologies, they are pivotal in advancing our understanding of mammary gland development and pathogenesis.

Recently, in 2023, Zou and his colleagues identified the role of the single-cell landscape in intratumoral heterogeneity and the immunosuppressive microenvironment in liver and brain metastases of BC (Zou et al., 2023). This finding was a significant breakthrough in BC research. In the same year, Xie and his co-authors discovered that single-cell histone chaperone patterns guide intercellular communication within the tumor microenvironment, contributing to BC metastases (Xie et al., 2023). In 2024, Liu and his colleagues reported that pan-cancer single-cell and spatial-resolved profiling revealed the immunosuppressive role of APOE + Macrophages in immune checkpoint inhibitor therapy (Liu et al., 2024).

### 1.4 SCS: multilayer analysis

Recent years have seen significant advancements in SCS technologies, enabling detailed analysis of the multilayered molecular states of individual cells (Hawsawi et al., 2022b). SCS can elucidate genomic, epigenomic, and transcriptomic heterogeneity within cellular populations, tracking changes across these levels. For instance, scRNA-seq has become widely utilized worldwide. However, RNA-seq of cancer tissue often captures transcripts from various cell types, including tumor cells, immune cells, fibroblasts, and endothelial cells. To accurately understand the transcriptomic status of such heterogeneous cell populations, specialized scRNA-seq techniques are employed.

As SCS technologies continue to evolve, research into molecular mechanisms at the single-cell level is advancing, driven by decreasing detection costs that are making these techniques more accessible. In 2017, Chen and colleagues developed a unique single-cell whole-genome amplification technique capable of more effectively detecting mutations and identifying CNVs with kilobase precision (Chen et al., 2017). Following this, in 2018, Casasent et al. (2018) introduced Topographic Single Cell Sequencing (TSCS), a technique that enables the precise determination of cellular spatial positions. This advancement has facilitated the mapping of cell distribution within tissues, yielding critical insights into cellular microenvironments and tissue architecture that were previously difficult to elucidate (Casasent et al., 2018). In the same year, using droplet microfluidics, Demaree et al. (2018) proposed a high-throughput and low-deviation SCS (SiC-seq) technique. This method separates, amplifies, and barcodes the genome of a single-cell, allowing for detailed analysis of genetic variations at the single-cell level (Demaree et al., 2018).

#### 1.4.1 Transcriptomic analysis

A recent study transcriptionally analyzed 3,71,223 cells from 34 tumors with mismatch repair deficiency and 28 tumors with mismatch repair proficiency in colorectal cancer and surrounding normal tissues (Pelka et al., 2021). This analysis led to the identification of spatially ordered immune-malignant cell networks and hubs of interacting malignant and immunological cells, which are critical for understanding the tumor microenvironment. Transcriptomic analysis has also been used to explore metastatic malignancies and the differences between primary tissues and micrometastases (Rath et al., 2024). Using patient-derived xenograft models of BC and scRNA-seq techniques, Davis and colleagues reported transcriptional heterogeneity in both primary tumors and micrometastases (Davis et al., 2020). They discovered that tissues with micrometastases exhibited unique characteristics associated with poor prognosis.

Furthermore, transcriptome analysis has been applied to investigate tumor evolution. In the study examining the transcriptome of mouse lung tumors from preneoplastic to advanced stages, including adenocarcinoma, scRNA-seq revealed the adoption of a high-plasticity cell state (HPCS) during carcinogenesis. This HPCS was characterized by a high rate of proliferation and differentiation potential. It was also found that this HPCS contributed to increased chemoresistance and poorer survival outcomes. These findings underscore the importance of understanding cellular plasticity and microenvironmental interactions in developing effective therapeutic strategies and improving patient outcomes.

#### 1.4.2 Epigenomic analysis

SCS, utilizing epigenomic tools that examine DNA methylation and chromatin states, can determine the lineage and differentiation status of specific cells (Lee et al., 2020). Several techniques are currently employed to profile DNA methylation, including single-cell whole-genome bisulfite sequencing (scWGBS), single-cell reduced representation bisulfite sequencing (scRRBS), single-cell bisulfite sequencing (scBS-seq), single-nucleus methylcytosine sequencing (snmC-seq), and single-cell combinatorial indexing for methylation (sci-MET) (Smallwood et al., 2014). These methods have proven effective in identifying reprogrammed regulatory elements present in metastatic disease. For example, epigenomic datasets created using bisulfite sequencing and ChIP-seq in normal prostate tissues, as well as in localized and metastasized prostate malignancies, have revealed critical insights into the epigenetic alterations associated with disease progression (Pomerantz et al., 2020).

To study open chromatin, several methodologies are available, including single-cell micrococcal nuclease sequencing (scMNase-seq), single-cell DNase sequencing (scDNaseseq), single-cell combinatorial indexing assays for transposase-accessible chromatin with sequencing (sci-ATAC-seq), and single-cell assays for transposase-accessible chromatin using sequencing (scATAC-seq) (Cooper et al., 2017). Additionally, the transcriptome and proteome of a single cell can be measured using specific techniques such as cellular indexing of transcriptomes and epitopes by sequencing (CITE-seq), the proximity ligation assay for RNA (PLAYR), the proximity extension assay/specific RNA target amplification (PEA/STA), and the RNA expression and protein sequencing assay (REAP-seq) (Lee et al., 2020). Zhou et al. (2020) developed a deep neural network-based approach, cTP-net, to predict surface protein abundances from scRNA-seq, further enhancing the utility of these datasets.

Epigenomic datasets, created through techniques such as bisulfite sequencing and ChIP-seq, can also reveal the existence of enhancers unique to metastasis, offering a deeper understanding of the molecular mechanisms underlying metastatic disease (Pomerantz et al., 2020).

#### 1.4.3 Genomic analysis

Single-cell whole-genome sequencing (scWGS) techniques have been employed to evaluate both germline and somatic mutations. These techniques involve the uniform amplification of genomic DNA from individual cells using methods such as multiple displacement amplification, multiple annealing and loop-based amplification cycles (MALBAC), degenerate oligonucleotide-primed PCR (DOP-PCR), and PicoPLEX (Kashima et al., 2020). scWGS has been particularly useful in exploring the relationship between aging, cancer, and B lymphocyte mutations (Zhang et al., 2019). Notably, research has shown that mutations in human B lymphocytes dramatically increasee with age, indicating genetic markers strongly associated with B cell malignancies.

In colon cancer research, scTrio-seq has been utilized to analyze the mutations, transcriptome, and methylome in both primary and metastatic tumors, providing comprehensive insights into colon cancer lineages (Bian et al., 2018). Recent advancements have also introduced single-cell exome sequencing (scExome-seq), which focuses on capturing and analyzing coding regions of the genome. This technique offers valuable insights into mutations affecting protein-coding genes and their potential impact on tumorigenesis (Gonçalves et al., 2021).

#### 1.4.4 Proteomic analysis

Proteomics has been approached through various methods, including RNA expression and protein sequencing assay (REAP-seq), cellular indexing of transcriptomes and epitopes by sequencing (CITE-seq), proximity ligation assay for RNA (PLAYR), and proximity extension assay/specific RNA target amplification (PEA/STA) (Lee et al., 2020). In a study involving approximately 400 human samples, extracellular vesicles and particles (EVPs) underwent proteomic analysis using the mass spectrometry technique known as parallel reaction monitoring (PRM). The findings demonstrated that EVP proteins could serve as biomarkers for cancer diagnosis and the identification of specific cancer types (Hoshino et al., 2020).

In clinical settings, SCS can be instrumental in identifying and developing disease-specific biomarkers. Researchers have developed a single-cell artificial intelligence model capable of learning and integrating biological function and imaging data. scRNA-seq remains a crucial technique for investigating transcriptome profiles, signal regulation, heterogeneity, evolution, and cell-cell communication (Zhang et al., 2022). Eight years after the pioneering publication of single-cell whole-transcriptome analysis, which marked a significant milestone in understanding cellular heterogeneity, the importance of scRNA-seq for clinical and translational medicine was widely acknowledged (Wang and Song, 2017). Single-cell transcriptome profiles provide new insights into monitoring gene signatures, cell markers, and intercellular processes, facilitating the discovery of function-, cell-, network-, and disease-specific biomarkers and offering a deeper understanding of their distinct roles and states.

The complexity of cell-cell communication, now accessible through scRNA-seq, presents opportunities for identifying biologically specific diagnostic markers and therapeutic targets. However, challenges remain in adapting bioinformatic tools, data analyses, and study designs from developmental biology to disease contexts. Combining scRNA-seq with single-cell DNA sequencing further reveals intra- and intercellular heterogeneity, temporal and spatial heterogeneity, clonal evolution in primary tumors, cell invasion clusters in early-stage cancers, metastatic spread trajectories, and the evolution of treatment resistance (Ellsworth et al., 2017). Recent advances in cancer research have highlighted the benefits and limitations of whole-transcriptome amplification methods, single-cell separation techniques, and bioinformatics analysis of clinical samples (Wang and Wang, 2017).

### 1.5 Single cell sequencing in BC research

SCS has revolutionized BC research by enabling the study of tumor heterogeneity, metastasis, and treatment response at an unprecedented level of detail. Traditional molecular methods, which often rely on bulk DNA or RNA from large cell populations, provide an average state of the cells, potentially masking signals from specific subpopulations. In contrast, single-cell genomic techniques offer the ability to resolve complex cellular mixtures within tumors, facilitating the identification of multiple clonal subpopulations and rare chemotherapy-resistant clones. For instance, scRNA-seq has been employed to analyze patient-derived xenograft models of BC, revealing transcriptional heterogeneity in both primary tumors and micrometastases. Notably, the latter exhibited a distinct profile associated with poor prognosis. This technology holds significant potential for enhancing the detection, progression monitoring, and prediction of therapeutic efficacy in BC (Mustachio and Roszik, 2022).

Moreover, SCS techniques such as Smart-seq, Smart-seq2, Quartz-Seq, and CEL-seq enable the measurement of full-length transcripts from isolated single cells, providing comprehensive insights into the transcriptomic status of specific cell populations. Recent studies have further showcased the power of SCS in identifying cellular states, gene networks, and tumor transformations, thereby generating valuable datasets for analyzing interacting cellular programs. These advancements underscore the transformative potential of SCS in unraveling the molecular complexities of BC.

### 1.6 Clinical applications of single cell sequencing in BC

scRNA-seq is a powerful tool that enables an in-depth exploration of cell state diversity and population heterogeneity. This method is particularly valuable for investigating the characteristics of various cell types within and surrounding breast tumors. By examining these intricacies at the single-cell level, scRNA-seq significantly advances our understanding of tumor proliferation, progression, and metastasis in BC (Chung et al., 2017). This enhanced comprehension lays the groundwork for personalized therapeutic strategies and the identification of novel biomarkers (Alzahrani et al., 2023). Ultimately, this technology facilitates the examination of individual cells, revealing the molecular complexities of BC, potentially revolutionizing our understanding of disease pathways, and leading to the development of individualized treatments and diagnostic approaches. Furthermore, scRNA-seq is crucial for studying transcriptome profiles, signal regulation, heterogeneity, evolution, and cell-cell communication in single-cell solutions (Zhang et al., 2022). Additionally, scRNA-seq has been instrumental in identifying rare cell subpopulations and tumor microenvironmental niches that contribute to therapy resistance and disease recurrence. This technology has already been employed in diverse applications within BC research.

### 1.7 Early detection and diagnosis

SCS technology promises to revolutionalize the early detection and diagnosis of BC. Traditional molecular methods, such as microarrays and next-generation sequencing, often struggle to accurately assess the complexity of solid tumors, which contain a heterogenous mixture of cells. This complexity can obscure signals from cancer cells, hindering precise analysis. Single-cell genomic methods, however, offer a solution by providing detailed insights into tumor complexity, identifying resistant clones, and monitoring CTCs. This capability significantly enhances detection, tracking of disease progression, and the prediction of therapeutic efficacy (Navin and Hicks, 2011).

Recent advancements in SCS techniques, such as single-cell combinatorial marker sequencing (SCI-seq) and topographic SCS (TSCS), enable the detection of somatic cell variations and the characterization of spatial features of tumor cell invasion (Mannarapu et al., 2021). Additionally, liquid biopsy approaches that employ SCS allow for early detection of cancer biomarkers from blood samples, offering a noninvasive method to monitor tumor dynamics and assess treatment responses. These innovations not only improve the accuracy and timeliness of BC diagnoses but also hold the potential for enhancing patient outcomes and survival rates.

### 1.8 Personalized treatment strategies

The development of personalized therapies has proven highly effective in BC treatment (Biancolella et al., 2021). With rapid advancement of sequencing technologies, SCS is set to further revolutionize personalized treatment strategies for BC patients. By leveraging individual genomic profiles, this technology can inform tailored therapeutic interventions, marking a significant shift toward precision oncology (Navin and Hicks, 2011). Single-cell genomic methods are particularly valuable for profiling rare cancer cells in clinical samples, monitoring CTCs, and detecting chemotherapy-resistant clones, thereby improving detection, progression tracking, and the prediction of therapeutic efficacy.

Furthermore, scRNA-seq has played a crucial role in understanding metastatic cancers by comparing primary tumors with micrometastases. This approach reveals distinct transcriptional profiles associated with poor prognosis, offering insights into potential therapeutic targets. For example, targeting pathways related to mitochondrial oxidative phosphorylation could help attenuate metastases in BC patients, underscoring the potential of SCS to inform targeted therapeutic approaches (Mustachio and Roszik, 2022). The integration of SCS data with multi-omic profiles further enhances the identification of novel therapeutic targets and biomarkers. However, implementing of SCS in clinical settings presents challenges, including high costs, complex data integration, and the need for advanced bioinformatics tools to translate findings into actionable treatment strategies.

### 1.9 Understanding the microenvironment of BC’s plasticity

Immune cells are crucial components of the tumor microenvironment (TME), with their phenotypes and features playing a pivotal role in understanding tumor progression and guiding immunotherapy strategies. TME reprogramming is primarily driven by the recruitment of immune cells and the rapid division of malignant cells. As a tumor grows, the microenvironment evolves, progressing from microscopic tumor foci to a palpable mass. T and B cells capable of destroying immune system cells invade early “indolent” tumor regions and become predominant. However, areas of tumor progression require increased cell proliferation and an environment that suppresses immunity. Immune-suppressive T cells release IL-17 to attract neutrophils and macrophages, which is crucial for increased myeloid cell infiltration and tumor spread (Sinha et al., 2021).

Azizi et al. (2018) found significant diversity among immune cell subtypes after analyzing CD45^+^ immune cells in eight BC patients using scDrop-seq. In breast TME, T-cell fractions account for the majority of immunological cells (21%–96%), with myeloid cells coming second (Azizi et al., 2018). Wagner et al. (2019) presented the single-cell atlas of the tumor and immune ecosystem of BC. They observed that ER− patients had the highest concentrations of Tregs, PD-L1 + TAMs, and PD-1 high CTLA-4 + CD38^+^ exhausted T cells when comparing immune microenvironments across different PAM50 subtypes. This suggests that the endocrine system remodels the BC microenvironment to support immunosuppressive function, explaining why immunotherapy may be more beneficial for ER− patients (Wagner et al., 2019). Sc-RNAseq studies of B-cell function in BC patients have shown that these cells respond to immune treatment and produce antibodies that enhance the effects of immune checkpoint blockade (ICB) and activate cytotoxic T cells (Hollern et al., 2019). Recent studies emphasize the role of tumor-infiltrating lymphocytes (TILs) in predicting patient outcomes and responses to immunotherapy. TILs, particularly those with activated or cytotoxic phenotypes, are associated with better prognosis and improved responses to checkpoint inhibitors (Guo et al., 2020).

Myeloid cells, including neutrophils, monocytes, and macrophages, contribute to tumor growth mainly through cytokine secretion and immune system suppression, though they exhibit considerable variability (Zilionis et al., 2019). Macrophages, the most prevalent myeloid cell type in tumor lesions, can adopt either an immunosuppressive M2 or proinflammatory M1 phenotype. M2-type genes, such as CD276, CD163, MS4A6A, and TGFB1, are extensively expressed in tumor-associated macrophages (Chung et al., 2017). ScRNA-seq has identified gene sets associated with M1 and M2 phenotypes, and converting M2 phenotype cells into M1 immune-activated macrophages has proven useful for inducing an immune response and enhancing ICB therapy (Sun et al., 2021). Cancer-associated fibroblasts (CAFs) constitute a significant portion of the tumor stroma. As tumors progress, healthy fibroblasts decrease, and malignant cells convert fibroblasts into CAFs, disrupting the healthy breast matrix. Due to their potent secretion and tissue attachment capabilities, CAFs are dispersed within tumor tissue rather than outside of it. Conventional CAFs primarily stimulate tumor growth through immunosuppressive mechanisms, neovascularization, and stromal remodeling (Mhaidly and Mechta-Grigoriou, 2020). Single-cell transcriptome analyses have identified Nidogen + perivascular fibroblasts and fibulin + stromal fibroblasts as the two primary functions of intratumoral heterogeneous CAFs (Bartoschek et al., 2018). Additionally, single-cell analyses have provided insights into the metastatic niche, revealing how tumor cells adapt to and grow in distant organs. Identifying specific gene expression patterns in metastatic cells can inform potential therapeutic targets to prevent metastasis (Chen et al., 2021). Wang et al. (2021) used Smart-seq2 and DNBelab C4 scRNA-seq to investigate the complex cancer microenvironment, measuring immune and nonimmune cell proportions, locations, and functions in colorectal cancer with hepatic metastases. Mei et al. (2021) employed scRNA-seq combined with whole-exome sequencing and transposase-accessible chromatin assays (scATAC-seq) to demonstrate molecular communication between cancer cells and immune cells. Chung et al. (2017) conducted a study involving 175 immune cells from 11 patients with various BC subtypes, including luminal A, luminal B, HER2, and TNBC. They classified these cells into three categories—T lymphocytes, B lymphocytes, and macrophages—by assessing their gene expression profiles at the single-cell level. Both T lymphocytes and macrophages exhibited features indicative of immunosuppression, with T cells showcasing a regulatory or exhausted phenotype and macrophages presenting an M2 phenotype (Chung et al., 2017). Despite the advances provided by scRNA-seq technology in elucidating immune cell diversity in the tumor microenvironment, further comprehensive investigations are needed. A deeper exploration of the impact of immune cell heterogeneity on the tumor microenvironment is crucial.

### 1.10 Understanding tumor heterogeneity in BC

Traditional bulk analysis often falls short in capturing the complete gene expression patterns of individual cells (Alzahrani et al., 2022). ScRNA-seq has been employed to comprehensively profile heterogeneous tumors in various BC subtypes, including TNBC. It has been suggested that a minor subpopulation of TNBC cells may not respond to conventional chemotherapy, potentially leading to metastasis. Identifying and characterizing these specific cells could provide insights into the nature of the disease and serve as a foundation for developing novel targets, personalized treatments, and diagnostic approaches, ultimately improving survival rates in TNBC (Ding et al., 2020).

Karaayvaz et al. (2018) utilized single-cell profiling, specifically scRNA-seq, to unveil subclonal heterogeneity and identify aggressive disease states in TNBC. Their scRNA-seq analysis of untreated primary TNBC tumors validated the presence of cellular heterogeneity and delineated five distinct clusters of cells. Notably, cluster 2 exhibited the highest proportion of high-cycling cells, indicating enhanced proliferation capability.

The metastatic process is a critical aspect of cancer progression, particularly in BC, where an increased count of CTCs is often associated with a higher risk of metastasis. The presence of CTCs is a significant prognostic factor for BC and correlates directly with patient survival rates. The activation of epithelial-mesenchymal transition in cancer cells leads to increased invasiveness and therapy resistance. As the disease progresses, the proportion of mesenchymal CTCs increases (Park et al., 2020). Researchers have been keenly investigating the role of CTCs in cancer progression, although current knowledge is limited due to difficulties in isolating a broad spectrum of CTC phenotypes.

CTCs exhibit significant heterogeneity and can be found both in clusters and as individual cells, comprising distinct phenotypic and genetic subpopulations (Menyailo et al., 2020). Recent technological advancements now enable the efficient isolation of CTCs and the application of highly accurate scRNA-seq. These advancements allow for the exploration of unique properties of CTCs beyond matching them to established tumor markers, offering insights into their survival mechanisms in the bloodstream and their potential to form distant metastases. Understanding these mechanisms is crucial for addressing metastases (Ting et al., 2014). This information provides valuable insights into the diverse characteristics of TNBC cells and may contribute to more targeted and effective therapeutic approaches.

## 2 Conclusion and future prospective

In conclusion, the application of SCS technologies has significantly transformed our understanding of BC, particularly by unraveling the complexities of tumor heterogeneity and the dynamic interactions within the tumor microenvironment. BC, known for its heterogeneity, presents challenges in developing effective and targeted treatments. The advent of SCS, especially in recent years, has provided a powerful tool for researchers to explore the intricacies of individual cells, offering insights into genomic, transcriptomic, epigenomic, and proteomic variations.

The emergence of SCS technologies marks a pivotal advancement in BC research, offering unparalleled opportunities for in-depth exploration and scientific breakthroughs. By revealing the intricate landscape of tumor heterogeneity and the dynamic interplay within the tumor microenvironment, SCS has revolutionized our understanding of this complex disease. The insights gained from single-cell analyses not only deepen our comprehension of BC biology but also present exciting prospects for pioneering diagnostic methodologies and personalized therapeutic interventions.
